# Aza-Amino Acids Disrupt β-Sheet Secondary Structures

**DOI:** 10.3390/molecules24101919

**Published:** 2019-05-18

**Authors:** Michael A. McMechen, Evan L. Willis, Preston C. Gourville, Caroline Proulx

**Affiliations:** Department of Chemistry, North Carolina State University, Raleigh, NC 27695-8204, USA; mamcmech@ncsu.edu (M.A.M.); elwilli5@ncsu.edu (E.L.W.); pcgourvi@ncsu.edu (P.C.G.)

**Keywords:** peptidomimetics, azapeptides, aza-amino acids, β-hairpin, β-sheet

## Abstract

Cα to N substitution in aza-amino acids imposes local conformational constraints, changes in hydrogen bonding properties, and leads to adaptive chirality at the nitrogen atom. These properties can be exploited in mimicry and stabilization of peptide secondary structures and self-assembly. Here, the effect of a single aza-amino acid incorporation located in the upper β-strand at a hydrogen-bonded (HB) site of a β-hairpin model peptide (H-Arg-Tyr-Val-Glu-Val-d-Pro-Gly-Orn-Lys-Ile-Leu-Gln-NH_2_) is reported. Specifically, analogs in which valine^3^ was substituted for aza-valine^3^ or aza-glycine^3^ were synthesized, and their β-hairpin stabilities were examined using Nuclear Magnetic Resonance (NMR) spectroscopy. The azapeptide analogs were found to destabilize β-hairpin formation compared to the parent peptide. The aza-valine^3^ residue was more disruptive of β-hairpin geometry than its aza-glycine^3^ counterpart.

## 1. Introduction

**Figure 1:**
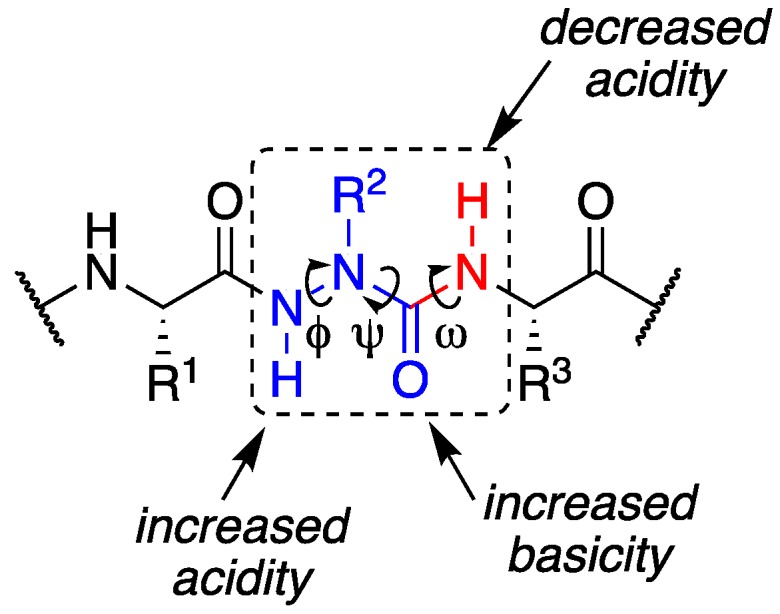
Aza-amino acid (in blue) creates one face with increased hydrogen bonding properties and one with reduced hydrogen bonding capacity.

β-sheets are common protein secondary structures that are often involved in protein-protein interactions and protein aggregation [1,2]. To understand factors governing folding, stability, and molecular recognition events involving β-sheets, water soluble β-hairpin model systems that are partially folded have been developed, consisting of two antiparallel β-strands connected by a reverse turn unit [3]. In the pursuit of protein-protein interaction (PPI) inhibitors, β-strand and β-sheet peptidomimetics have been explored using a variety of unnatural scaffolds [4]. When mimicking β-strands in particular, the hydrogen bonding pattern of a natural peptide is ideally replicated by the artificial templates to maintain cross-strand interactions, yet surface exposed hydrogen bonding sites should be minimized to prevent uncontrolled aggregation, creating a so-called “blocking strand” [5,6,7]. Here, we describe the effect of a single Cα substitution for a nitrogen at the Val^3^ position of a model β-hairpin peptide. The aza-amino acid residue was envisioned to provide two distinct faces with divergent hydrogen bonding capabilities, while retaining side chain position and chemistry (Figure 1).

**Figure 2:**
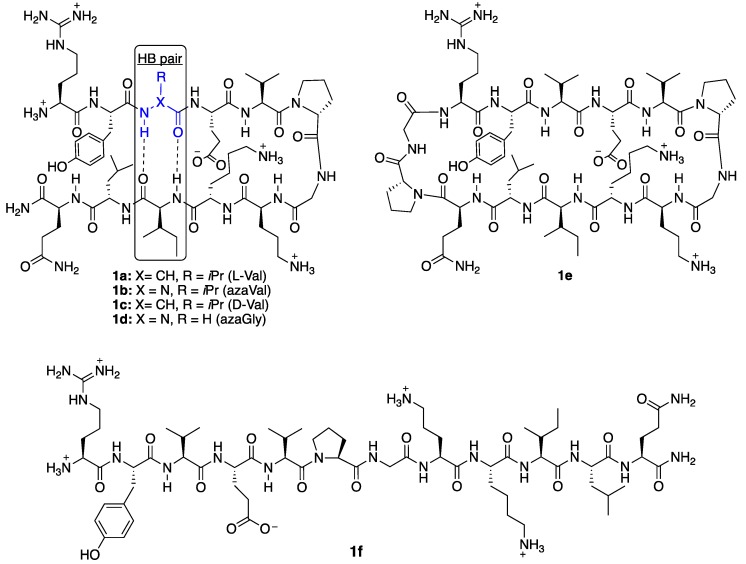
β-hairpin model peptide **1a** [8], derivatives **1b–d**, and controls **1e–f** [8] under study.

Aza-amino acids [8] have been used to promote β-turn secondary structures and hyperstable collagen peptide mimics [9,10] via a combination of hydrogen bonding and backbone dihedral angle modulation. Their effect on β-hairpin stability has yet to be quantified within β-strand regions. In aza-amino acids, perpendicular lone pair orientation between adjacent nitrogen and urea planarity typically reinforce backbone dihedral angles that fall within the range of β-turns and polyproline type II secondary structures [8]. Although this may prevent adoption of extended β-strand geometry [11], computational studies have set the energy barrier for rotation from the twisted conformer (φ = 90°) to the extended conformer (φ = 180°) to be < 1 kcal/mol within 1,2-diformylhydrazine in the *Z*,*Z* configuration as an azaGly model [12]. Two favorable intramolecular hydrogen bonds are proposed to stabilize the 180° orientation. Although addition of side chain chemistry in aza-amino acids removes an intramolecular hydrogen bond with a neighboring residue, cross-strand hydrogen bonding and side chain; side chain interactions in a β-hairpin may provide added stability to the extended conformer. Substitution of Val^3^ for aza-valine^3^ was thus examined in a well-studied β-hairpin model system for which Nuclear Magnetic Resonance (NMR)-based quantification of β-hairpin folding have been established, first reported by Gellman et al. [13,14]: H-Arg-Tyr-Val-Glu-Val-d-Pro-Gly-Orn-Lys-Ile-Leu-Gln-NH_2_ (**1a**). Valine^3^ is situated in the middle of the upper β-strand at a hydrogen bonded (HB) site (Figure 2, in blue), equally removed from the d-Pro-Gly turn region and the *N*-terminus. In addition to azaVal^3^ analog **1b**, d-Val^3^ control **1c**, azaGly^3^ analog **1d**, and previously described [14] positive control cyclic peptide **1e**, and negative control l-Pro^6^-Gly^7^ peptide **1f**, all were synthesized and studied (Figure 2). Since the first incorporation of azaVal to give an azapeptide angiotensin analog [15], considerable interest has been generated in the synthesis and conformational properties of this aza-amino acid.

## 2. Results and Discussion

### 2.1. Synthesis

**Scheme 1:**
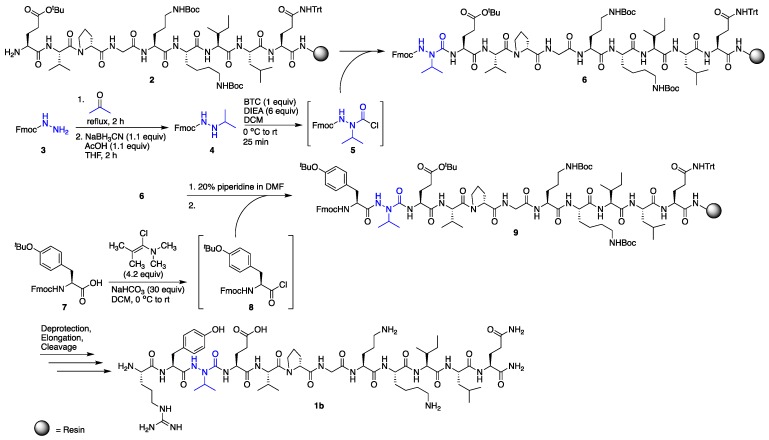
Synthesis of azapeptide **1b**.

Solid-phase peptide synthesis was performed on Rink amide resin, using standard Fmoc procedures for all l- and d-amino acid coupling and deprotection cycles [16]. Incorporation of azaVal^3^ for the synthesis of **1b** was accomplished via activation of *N*’-isopropyl-fluorenylmethyl carbazate [17] using bis-(trichloromethyl)carbonate (BTC) as the activating agent (Scheme 1) and as a safer alternative to phosgene. Although submonomer azapeptide synthesis protocols [18] were considered for the installation of aza-valine to circumvent the solution-phase synthesis and activation of hydrazine building blocks, *N*-alkylation of resin-bound semicarbazone intermediates with sterically hindered secondary alkyl halides has typically resulted in lower conversions [19]. In our hands, incorporation of aza-valine via *N*’-alkyl fluoren-9-ylmethyl carbazate activation with triphosgene afforded azapeptide **6** as the major product in 77% crude purity after a single overnight coupling reaction. In contrast, in accordance with previous literature, coupling to the aza-amino acid to give **9** was challenging due to the decreased nucleophilicity of semicarbazides [8] and steric hindrance from the branched aza-valine side chain. In the past, coupling to aza-valine and other aza-amino acids with branched side chains has been accomplished using *N*,*N*’-dicyclohexylcarbodiimide (DCC) [15], diisoproylcarbodiimide (DIC) [19,20], and BTC [21]. Here, the coupling of Fmoc-Tyr(^t^Bu) to resin-bound azaV-EVpGOKILQ using BTC and 2,4,6-collidine in tetrahydrofuran (THF) provided **9** in only 33% conversion by liquid chromatography-mass spectrometry (LC-MS) analysis, with 21% uncoupled semicarbazide and 28% isocyanate or hydantoin byproduct [22] (Table 1, entry 1). In the context of β-hairpin synthesis, the already difficult coupling to aza-Val may be rendered even more challenging by folding or aggregation on-resin [23]. Attempts employing a LiCl salt additive to the BTC coupling solution to disrupt possible on-resin aggregation [24] were less successful than efforts to optimize the coupling reagent (Table 1, entries 2–5).

Recent successes using benzotriazol-1-yloxytris(pyrrolidinophosphonium-hexafluorophosphate (PyBOP)/*N*,*N*-diisopropylethylamine (DIEA) [25] and (1-cyano-2-ethoxy-2-oxoethylidenaminooxy)dimethylaminomorpholino-carbenium hexafluorophosphate (COMU)/DIEA [26,27] in difficult couplings to aza-amino acids led us to try these activating agents; however, **9** was obtained in low conversions in both cases (6–13%, Table 1, entries 2 and 3). We next investigated *N,N,N′,N′*-tetramethylchloroformamidinium hexafluorophosphate (TCFH)/*N*-methylimidazole (NMI) (Table 1, entry 4), which was recently developed [28] to couple sterically hindered carboxylic acids with non-nucleophilic aniline derivatives and sterically hindered amino acids, including valine. Gratifyingly, these conditions provided **9** in high conversion (89%) with little byproduct formation and no detectable starting material (Table 1, entry 4), although appearance of two peaks with identical masses by LC-MS suggested possible epimerization. Alternatively, the use of 1-chloro-*N*,*N*,2-trimethyl-1-propenylamine (Ghosez’s reagent) [29] (Table 1, entry 5) provided the desired elongated azapeptide **9** as a single peak by LC-MS analysis in 67% conversion, with no uncoupled semicarbazide detected. Using this procedure for coupling onto azaVal, azapeptide **1b** was obtained in 53% crude purity, following deprotection, elongation, and cleavage from the resin. Purification of the crude azapeptide by reverse phase – high performance liquid chromatography (RP-HPLC) afforded **1b** in 8% yield (see Supporting Information).

Activation of *N*-Fmoc-hydrazine with phosgene equivalents was previously shown to lead to the formation of a cyclic oxadiazolone [30]. Although ring-opening of this heterocycle to afford the desired Fmoc-protected azaGly-terminated peptides may be possible [30], activation and coupling of aza-glycine using these conditions has been reported to be problematic in certain cases [31]. Here, aza-glycine installation was accomplished by activating fluoren-9-methyl carbazate with *N,N′*-disuccinimidyl carbonate (DSC) as the activating agent instead of BTC, which afforded the desired azapeptide in higher yields [32]. Fmoc-Tyr(*^t^*Bu)-OH activation and coupling to the resin-bound aza-glycine-terminated peptide was accomplished using BTC/2,4,6-collidine in THF and required no further optimization.

### 2.2. Conformational Analysis

**Figure 3:**
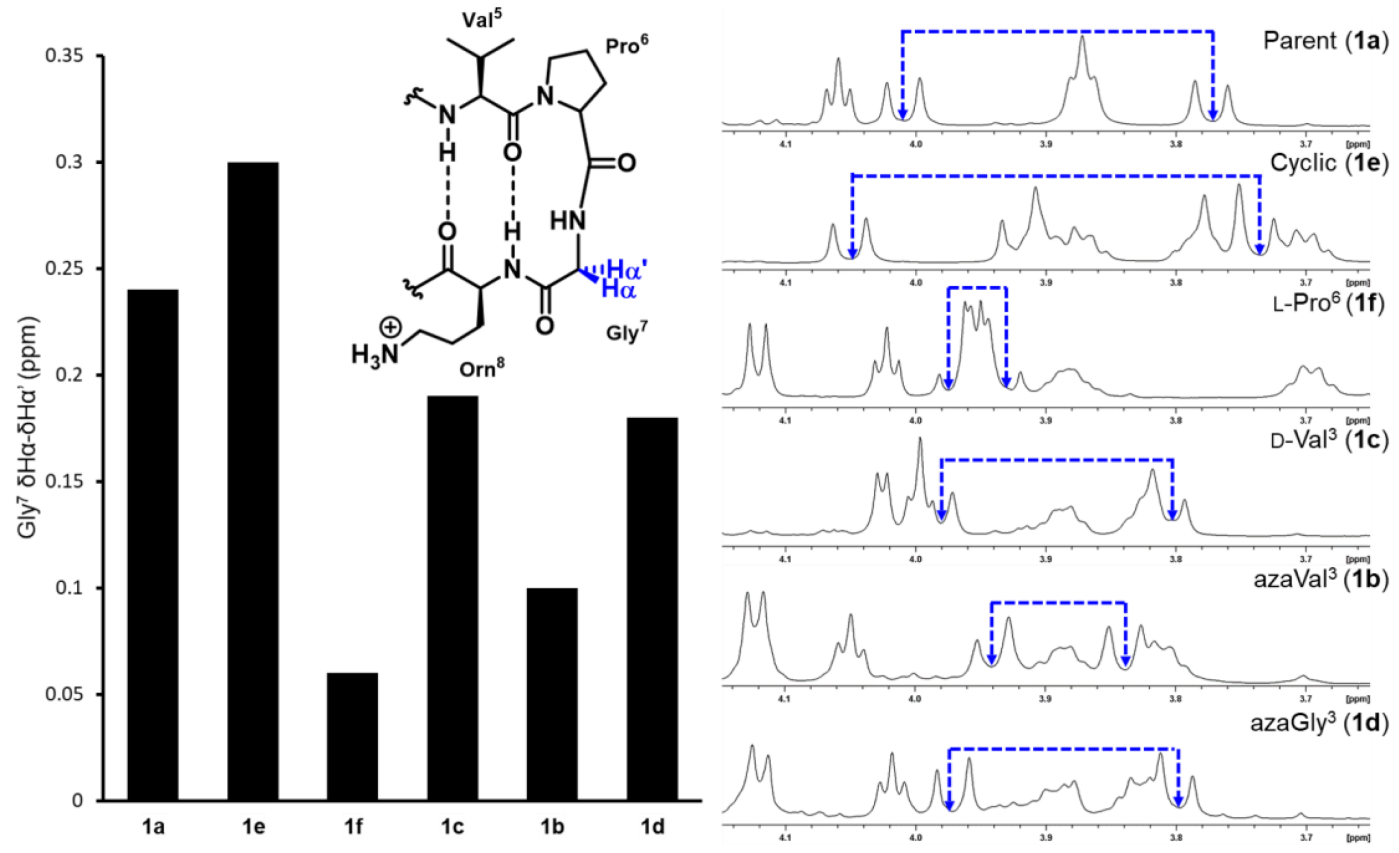
Diastereotopic splitting of Gly^7^ Hα chemical shifts.

β-Hairpin folding was first assessed by measuring the extent of diastereotopic Gly^7^ Hα splitting in the turn region for each analog and comparing it to the fully folded cyclic control (Equation (1)), as previously described [33]. Using this method, the relative stability of each β-hairpin analog can be quickly compared as the glycine signals lie in a distinct region of the spectra (Figure 3, Table 2).

Fraction folded = (∆δ_Gly Obs_)/(∆δ_Gly 100_) (1)

Replacing valine^3^ for an aza-valine residue in analog **1b** caused a significant decrease in β-hairpin stability, reflected by the much smaller Gly^7^ δHα–δHα’ value (Figure 3). Potential cross-strand hydrogen bonding and side chain - side chain interactions between azaVal^3^ and Ile^10^ appear to be insufficient to stabilize the extended conformation in aza-valine^3^, which likely adopts a twisted conformation (φ = 90°) that disrupts β-hairpin formation. Because aza-amino acids may exhibit adaptive chirality at Nα, the aza-valine residue could be achiral or exhibit either l or d-like chirality [34], which could in turn affect β-hairpin stability. We synthesized d-Val^3^ analog **1c** as a control and found its Gly^7^ δHα–δHα’ value to be larger than that of aza-Val^3^ analog **1b**, yet smaller than the parent peptide **1a**. While changing the stereochemistry of the valine^3^ residue from l to d decreased β-hairpin stability overall, this substitution was found to be less disruptive than the backbone Cα to Nα substitution in the aza-valine residue. As such, the destabilizing effect of the aza-valine residue cannot be solely attributed to the loss of a defined stereocenter.

Given that the extended conformation in aza-glycine vs. aza-valine residues may be more favorable due to additional intramolecular hydrogen-bonding interactions (*vide supra*), the synthesis and conformational analysis of azaGly^3^ analog **1d** was also pursued. Despite the absence of an isopropyl side chain, the aza-glycine substitution was found to be less detrimental to β-hairpin stability compared to the aza-Val^3^ analog **1b**; however, β-hairpin folding was still decreased relative to the parent peptide **1a**. The disruptive effects of the azaGly^3^ substitution are similar those observed in the d-Val^3^ analog using Gly^7^ diastereotopic Hα splitting measurements (Figure 3) [35].

It should be noted that glycine splitting measurements have been found to overestimate β-hairpin folding in sequences that use d-Pro-Gly turn units [36]. Downfield chemical shifts in CHα protons relative to unfolded peptide for residues away from the turn region, especially those situated at HB sites, can be used to quantify the degree of β-hairpin folding more reliably according to Equation (2), where δ_0_ and δ_100_ are the chemical shift values for the unfolded analog **1f** and the cyclic control **1e**, respectively.

Fraction folded = (δ_Obs_ − δ_0_)/(δ_100_ − δ_0_) (2)

Specifically, the downfield chemical shifts of Val^3^, Ile^10^, and Orn^8^ have been found to be more reliable and are typically used to measure folded fractions for this β-hairpin model peptide and its derivatives [14]. Here, because the Cα of Val^3^ is substituted by a nitrogen in our azapeptide analogs, we omitted this residue from our studies (Table 2). The CHα chemical shift values for Ile^10^ are particularly informative because this residue sits in the middle of the lower strand and is positioned to form hydrogen bonds with the variable residue in the third position; Orn^8^ is situated closer to the turn region. Based on the chemical shift values for Ile^10^, aza-Val^3^ peptide 1b is completely unfolded, in contrast to the partially folded structure predicted based on glycine^7^ Hα splitting measurements (Figure 3, Table 2). Considering that diastereotopic splitting of Gly^7^ Hα overestimate folding in β-hairpin model peptides that have d-Pro-Gly turn units, incorporation of azaVal^3^ is likely completely disrupting the β-hairpin fold. In contrast, the azaGly^3^ analog 1d which lacks side chain functionality was ~50% folded based on the Ile^10^ downfield Hα chemical shifts, and exhibited a greater percentage of folding relative to both the aza-valine (1a) and d-valine (1c) analogs. The additional potential for azaGly to intra- and/or inter-strand hydrogen-bond may stabilize the extended conformer. The % folding values calculated from Orn^8^ chemical shifts reinforced the conclusion that the aza-Valine analog 1b was the least folded of all analogs, yet suggested similar degrees of folding for the d-Val (1c) and the azaGly (1d) analogs.

## 3. Materials and Methods

### 3.1. General

Polystyrene Rink Amide resin (0.78 mmol/g) and Fmoc-glycine-2-chlorotrityl resin (200–400 mesh, 0.493 mmol/g) were purchased from Protein Technology Inc™ and Chem-Impex Int’l, Inc, respectively, and the manufacturer’s reported loading of the resin was used in the calculation of the yields of the final products. Solid phase peptide synthesis was performed using an automated Biotage Syro Wave^TM^ peptide synthesizer in 10 mL parallel reactors with polytetrafluoroethylene (PTFE) frits. Incorporation of aza-amino acids and further elongations were performed manually in disposable filter columns with 20 µM polyethylene (PE) frit filters and caps purchased from Applied Separations (cat # 2413 and 2416 for 3 mL and 6 mL filter columns, respectively) with gentle agitation on a Thermo Fisher vortex mixer equipped with a microplate tray. Solution draining and washing of the resin was accomplished by connecting the filter columns to a water aspirator vacuum via a waste trap. Analytical LC-MS analyses were performed using an Agilent Technologies 1260 Infinity II series LC-MS Single Quad instrument with ESI ion-source and positive mode ionization, equipped with a 5 µM, 150 × 4.6 mm C18 Vydac column purchased from Mac-Mod Analytical, Inc (cat # 218TP5415). A flow rate of 0.5 mL/min and 5–95%, 20–80%, or 12–60% gradients of CH_3_CN [0.1% trifluoroacetic acid (TFA)] in water (0.1% TFA) over 12 min (total run time = 22 min) were used for all LC-MS analyses. Peptides were purified on a preparative HPLC (Agilent 218 purification system) using a preparative column (10–20 µM, 250 mm × 22 mm, C18 Vydac column, cat # 218TP101522) at a flow rate of 10 mL/min with gradients of CH_3_CN [0.1% trifluoroacetic acid (TFA)] in water (0.1% TFA) over 30 min (total run time = 60 min).

### 3.2. Reagents

Amino acids, *N,N′*-diisopropylethylamine (DIEA), *N,N′*-Disuccinimidyl carbonate (DSC), Fmoc chloride, (1-cyano-2-ethoxy-2-oxoethylidenaminooxy)dimethylamino-morpholino-carbenium hexafluorophosphate (COMU), chloro-*N,N,N′,N′*-tetramethylformamidinium hexafluorophospate (TCFH) and triphosgene (BTC) were purchased from Chem Impex Int’l, Inc. Reagents such as piperidine, lithium chloride, sodium cyanoborohydride (NaBH_3_CN), triisopropylsilane (TIPS), sodium acetate-d_3_, acetic acid-d_4_, and D_2_O were purchased from Sigma Aldrich. Reagents such as hydrazine monohydrate and 2,4,6-collidine were purchased from Alfa Aesar. Trifluoroacetic acid, glacial acetic acid, sodium bicarbonate, and solvents were purchased from Fisher. Reagents including HBTU and PyBOP were purchased from Oakwood Chemical. 1-Methylimidazole (NMI) and 1-chloro-*N,N*,2-trimethylpropenylamine (Ghosez’s Reagent) were purchased from Acros Organics.

### 3.3. 9-Fluorenylmethoxycarbonyl (Fmoc)-Based Solid Phase Peptide Synthesis (SPPS): Fmoc Deprotection and HBTU Couplings

Peptide syntheses were performed under standard manual conditions on an automated shaker or using a Biotage Syro Wave^TM^ peptide synthesizer using Polystyrene Rink Amide resin (0.78 mmol/g). Couplings of amino acids (3 equiv) were performed in DMF using HBTU (3 equiv) as coupling reagent and DIEA (6 equiv) as base. Fmoc deprotections were performed initially by treating the resin with 20% piperidine in DMF (*v/v*) for 5 min, followed by treatment with a fresh solution of 20% piperidine in DMF (*v/v*) for 15 min. Resin was washed after each coupling with DMF (3 × 1 mL) and deprotection reaction with DMF, MeOH, and CH_2_Cl_2_ (2 × 1 mL). Prior to cleavage from the resin or storage, resin was washed with CH_2_Cl_2_ (3 × 1 mL).

### 3.4. Test Cleavages of Resin-Bound Peptides

A small amount of resin (1–5 mg) was washed with CH_2_Cl_2_ to remove traces of DMF, drained, and treated with a freshly made solution of TFA/H_2_O/TIPS (90:5:5, *v/v/v*, 0.5 mL) for 2 h at room temperature. The cleavage solution was collected by filtering the resin through a disposable, PE fritted cartridge. The filtrate was evaporated to dryness and the crude peptide was precipitated twice with cold ether (4 mL) followed by decanting. The pellet (crude peptide sample) was dissolved in 1:1 MeCN/H_2_O *v/v* (1 mg/mL) and subjected to LC-MS analysis.

### 3.5. N′-Isopropyl-fluoren-9-ylmethyl Carbazate (**4**)

Synthesized according to literature procedures [12,16]. Briefly, a solution of Fmoc-Cl (2.00 g, 7.73 mmol) in 120 mL of CH_3_CN was added dropwise over 2 h at 0 °C to a solution of excess hydrazine hydrate (3.8 mL, 78.0 mmol, 10 equiv) in 26 mL of CH_3_CN/H_2_O (1:1, *v/v*). The solution was warmed to room temperature and left stirring for 12 h, prior to being concentrated in vacuo and filtered. The resulting solid was washed with water, followed by hexanes, to give 1.92 g of white solid in 98% yield. The resulting 9H-fluoren-9-ylmethyl hydrazinecarboxylate **3** (1.92 g, 7.55 mmol) was suspended in acetone (50 mL) and heated at reflux for 2 h. Acetone was evaporated in vacuo and the hydrazone intermediate was dissolved in THF (50 mL), treated with acetic acid (0.48 mL, 8.39 mmol, 1.1 equiv) and NaBH_3_CN (521 mg, 8.31 mmol, 1.1 equiv), and stirred for 2 h. The volatiles were removed and the crude was dissolved in EtOAc (100 mL), washed with 1 M aqueous KHSO_4_ (4 × 50 mL) and brine (2 × 50 mL), dried over Na_2_SO_4_, and concentrated to give a white solid. The obtained product was dissolved in EtOH and heated for 1 h, followed by evaporation of EtOH to yield *N*’-isopropyl-fluoren-9-ylmethyl carbazate **4** as a white solid (1.32 g, 59% yield) after column chromatography using a 20–100% gradient of EtOAc in hexanes. NMR (CDCl_3_) spectra matched literature values [12].

### 3.6. Incorporation of Aza-Valine on the Solid Phase

*N*’-isopropyl-fluoren-9-ylmethyl carbazate **4** (138 mg, 0.466 mmol, 3 equiv) was dissolved in dry CH_2_Cl_2_ (1 mL) and cooled to 0 °C. A solution of bis(trichloromethyl)carbonate (BTC, 46.4 mg, 0.156 mmol, 1 equiv) dissolved in dry CH_2_Cl_2_ (1 mL) and cooled to 0 °C was added dropwise to the *N*’-isopropyl-fluoren-9-ylmethyl carbazate suspension. The reaction mixture was left stirring at 0 °C for 5 min, allowed to warm to room temperature, and stirred for another 25 min. DIEA (0.163 mL, 0.930 mmol, 6.0 equiv) was added to the solution and the solution was stirred for 5 min prior to being transferred to a SPPS cartridge containing resin-bound peptide **2** (200 mg, 0.156 mmol) swollen in CH_2_Cl_2_. The SPPS cartridge was left shaking for 12 h, after which the solution was drained, and the resin was washed with CH_2_Cl_2_ (3 × 2 mL).

### 3.7. Coupling to AzaV-EVpGOKILQ

#### 3.7.1. BTC Coupling

Fmoc-Tyr(*^t^*Bu)-OH (53.8 mg, 0.117 mmol, 3.0 equiv) was dissolved in dry THF (0.3 mL), cooled to 0 °C, and treated dropwise with a cold solution of BTC (11.5 mg, 0.039 mmol, 1.0 equiv) in dry THF (0.3 mL). The reaction mixture was left stirring at 0 °C for 15 min, before adding 2,4,6-collidine (0.052 mL, 0.39 mmol, 10.0 equiv). The solution was stirred at 0 °C for 2 min, allowed to warm to room temperature, and stirred another 5 min. The activated amino acid was then transferred to a SPPS cartridge containing the resin-bound azapeptide (50 mg, 0.039 mmol), previously swollen in dry THF. The resin was left shaking at room temperature overnight, then washed with DMF (2 × 0.5 mL), MeOH (2 × 0.5 mL), and THF (2 × 0.5 mL).

#### 3.7.2. PyBOP Coupling

Fmoc-Tyr(*^t^*Bu)-OH (53.8 mg, 0.117 mmol, 3.0 equiv) and PyBOP (60.9 mg, 0.117 mmol, 3.0 equiv) were each dissolved in DMF (0.3 mL) and cooled to 0 °C separately. The PyBOP solution was then added dropwise to the solution of Fmoc-Tyr(*^t^*Bu)-OH and the reaction mixture was stirred at 0 °C for 5 min. DIEA (0.0408 mL, 0.234 mmol, 6.0 equiv) was added to the solution and the reaction was allowed to warm to room temperature before being transferred to a SPPS cartridge containing the resin-bound azapeptide (50 mg, 0.039 mmol), previously swollen in DMF. The resin was shaken at room temperature overnight, then washed with DMF (2 × 0.5 mL), MeOH (2 × 0.5 mL), and CH_2_Cl_2_ (2 × 0.5 mL).

#### 3.7.3. COMU Coupling

Fmoc-Tyr(*^t^*Bu)-OH (89.6 mg, 0.195 mmol, 5.0 equiv) and COMU (83.5 mg, 0.195 mmol, 5.0 equiv) were each dissolved in 0.3 mL of DMF and cooled to 0 °C separately. The COMU solution was added dropwise to the solution of Fmoc-Tyr(*^t^*Bu)-OH and the reaction mixture was stirred at 0 °C for 5 min. DIEA (0.0504 mL, 0.39 mmol, 10.0 equiv) was added and the reaction was warmed to room temperature before being transferred to a SPPS cartridge containing the resin-bound azapeptide (50 mg, 0.039 mmol), previously swollen in DMF. The resin was shaken at room temperature overnight, then washed with DMF (2 × 0.5 mL), MeOH (2 × 0.5 mL), and CH_2_Cl_2_ (2 × 0.5 mL).

#### 3.7.4. NMI/TCFH Coupling

Fmoc-Tyr(*^t^*Bu)-OH (53.8 mg, 0.117 mmol, 3.0 equiv) was dissolved in dry CH_2_Cl_2_ (0.3 mL), to which *N*-methyl imidazole (0.028 mL, 0.351 mmol, 9 equiv) was added before cooling the solution to 0 °C. A solution of TCFH (32.8 mg, 0.117 mmol, 3 equiv) in dry CH_2_Cl_2_ (0.3 mL), cooled to 0 °C, was added to the Fmoc-Tyr(*^t^*Bu)-OH and NMI solution in a single portion and the reaction was immediately transferred to a SPPS cartridge containing the resin-bound azapeptide (50 mg, 0.039 mmol), previously swollen in dry CH_2_Cl_2_. The resin was shaken at room temperature overnight, then washed with DMF (2 × 0.5 mL), MeOH (2 × 0.5 mL), and CH_2_Cl_2_ (2 × 0.5 mL).

#### 3.7.5. Ghosez Coupling

NaHCO_3_ (98.3 mg, 1.17 mmol, 30 equiv) was added to the resin-bound azapeptide (50 mg, 0.039 mmol) pre-swelled in dry CH_2_Cl_2_ (0.2 mL) and shaken for 15 min. During that time, Fmoc-Tyr(*^t^*Bu)-OH (53.8 mg, 0.117 mmol, 3.0 equiv) was dissolved in dry CH_2_Cl_2_ (0.4 mL) and cooled to 0 °C. Ghosez’s reagent (0.0217 mL, 0.164 mmol, 4.2 equiv) was added dropwise to the cooled solution of Fmoc-Tyr(*^t^*Bu)-OH and stirred for 5 min. The reaction was allowed to warm to room temperature for 5 min, prior to being transferred to the SPPS cartridge containing NaHCO_3_ in CH_2_Cl_2_. The resin was shaken at room temperature overnight. The resin was washed with DMF (2 × 0.5 mL), MeOH (2 × 0.5 mL), and CH_2_Cl_2_ (2 × 0.5 mL).

### 3.8. Incorporation of Aza-Glycine on the Solid Phase

*N,N′*-Disuccinimidyl carbonate (DSC) (120 mg, 0.466 mmol, 3.0 equiv) was added to a solution of 9H-fluoren-9-ylmethyl hydrazinecarboxylate **3** (123 mg, 0.484 mmol, 3.1 equiv) in DMF and stirred for 5 min. The solution was then transferred to resin-bound peptide **2** (200 mg, 0.156 mmol) swollen in DMF and left shaking for 12 h. The solution was drained and the resin was washed with DMF (3 × 2 mL).

### 3.9. Coupling to azaG-EVpGOKILQ

Fmoc-Tyr(*^t^*Bu)-OH (22.2 mg, 0.0484 mmol, 3.1 equiv) was dissolved in dry THF (0.3 mL), cooled to 0 °C, and treated dropwise with a cold solution of BTC (4.63 mg, 0.0156 mmol, 1.0 equiv) in dry THF (0.2 mL). The reaction mixture was left stirring at 0 °C for 15 min before adding 2,4,6-collidine (0.0206 mL, 0.156 mmol, 10.0 equiv). The solution was stirred at 0 °C for 5 min. The reaction was allowed to warm to room temperature prior to transferring the activated amino acid to a SPPS cartridge containing the resin-bound azapeptide (20 mg, 0.0156 mmol) previously swollen with dry THF. The reaction was left shaking at room temperature overnight, then washed with DMF (2 × 0.5 mL), MeOH (2 × 0.5 mL), and THF (2 × 0.5 mL).

### 3.10. Synthesis of Cyclic Peptide 1e

The linear precursor H_2_N-Arg(Pbf)-Tyr(*^t^*Bu)-Val-Glu(O^t^Bu)-Val-pro-Gly-Orn(Boc)-Lys(Boc)-Ile-Leu-Gln(Trt)-pro-Gly-OH was synthesized on Fmoc-glycine-2-chlorotrityl resin (200–400 mesh, 0.493 mmol/g loading) according to standard SPPS protocols described above. Liberation of the side chain protected peptide from solid support was conducted using a freshly prepared solution of hexafluoroisopropanol in CH_2_Cl_2_ (20% *v/v*), the procedure was repeated, solutions were combined, and volatiles were removed in vacuo. The protected linear peptide was dissolved in DMF (15 mL) and a solution of HBTU (41.2 mg, 0.109 mmol, 6 equiv) and HOBt^●^H_2_O (16.6 mg, 0.109 mmol, 6 equiv) in DMF (1 mL) was added dropwise, followed by an addition of DIEA (0.0494 mL, 0.283 mmol, 15 equiv) in DMF (1 mL). The reaction was allowed to proceed overnight under N_2_ atmosphere. DMF was removed in vacuo and the cyclic side chain protected peptide was carried forward to global deprotection. A fresh 90:5:5 (v/v/v) solution of TFA: H_2_O: TIPS was prepared and ~10 mL were added to the dried peptide. The reaction was stirred for 2 h at room temperature, followed by the removal of all volatiles in vacuo. Cold ether precipitation was performed followed by dissolution of the cyclic peptide in 20% MeCN in water (3 mL). The crude peptide sample was purified by RP-HPLC according to protocols described in General Methods. 

### 3.11. Full Cleavage and Purification of (Aza)Peptides

The resin-bound (aza)peptide was washed with CH_2_Cl_2_ to remove traces of DMF, drained, transferred into a 20 mL scintillation glass vial, and treated a freshly made solution of TFA/H_2_O/TIPS (90:5:5, v/v/v, 5 mL). The vial was capped and agitated for 2 h at room temperature on an orbital shaker. The cleavage mixture was filtered through a disposable, PP fritted cartridge into a 50 mL falcon tube containing cold ether (~20 mL). The ether was decanted following centrifugation at 8000 rpm for 2 min. The peptide pellet was suspended in 1:9 MeCN/H_2_O *v/v* (5 mL). The sample was frozen and lyophilized. The crude (aza)peptpide was redissolved in a MeCN/H_2_O solvent mixture and purified using reverse-phase HPLC. See Supporting Information for characterization data.

### 3.12. NMR Spectroscopy

Lyophilized (aza)peptides were diluted to concentrations of 3–4 mM in D_2_O buffered with 50 mM NaOAc-d3 buffer at pH 4.2 (uncorrected), pH adjusted with AcOH-d3 [36]. All NMR spectra were collected at the Molecular Education, Technology, and Research Innovation Center (METRIC) at NC State University on a Bruker NEO 700 MHz instrument with a TCI cryoprobe at 5 °C. 1D spectra were collected with 32K data points and at least 32 scans and water suppression with a pre-saturation pulse or with a 1D-1H NOESY. 2D-experiments were collected on the same instrumentation, using the standard Bruker ROESY, COSY, and TOCSY pulse sequences with presaturation. All 2D experiments were done with eight scans, 2048 points in the first dimension, and 256 or 512 in the indirect dimension. TOCSY spectra were collected with 80 ms spin-lock and ROESY spectra were collected with a mixing time of 200 ms. Spectral analysis was performed with TopSpin 4.0.5 software.

## 4. Conclusions

Aza-amino acids have previously been shown to stabilize β-turn secondary structures and impart hyperstability to self-assembling collagen peptide mimics. Here, we expand the folding rules for aza-amino acids beyond turns and polyproline type II helices and characterize their effect on β-hairpin stability when incorporated into the β-strand region. Using NMR spectroscopy, we demonstrate that a valine to aza-valine substitution significantly disrupts β-hairpin folding, based on both glycine splitting measurements and CHα proton shifts at the Orn^8^ and Ile^10^ residues relative to cyclic and unfolded controls. Aza-glycine incorporation at the same position was found to retain some folding, despite loss of side chain chemistry, which may be due to additional hydrogen bonding. Overall, our findings support earlier computational hypothesis that aza-amino acids are not well tolerated within β-sheet secondary structures.

## Figures and Tables

**Table 1:** Optimization of coupling to resin-bound aza-VEVpGOKILQ.

Entry	Coupling Reagent	Equiv.	Base	Equiv.	Solvent	% Coupled	% Uncoupled	% Isocyanate/Hydantoin Byproduct [22]
1	BTC	1	2,4,6-collidine	10	THF	33	21	28
2	PyBOP	3	DIEA	6	DMF	6	35	41
3	COMU	5	DIEA	10	DMF	13	17	53
4	TCFH^a^	3	NMI	9	CH_2_Cl_2_	89^a^	0	10
5	Ghosez	4.2	NaHCO_3_	30	CH_2_Cl_2_	67	0	22

^a^ Possible epimerization was detected by LC-MS analysis.

**Table 2:** % folding based on glycine^7^ splitting and Hα chemical shifts for Orn^8^ and Ile^10^.

Peptide	% Folding			
	Gly^7^ δHα–δHα’	Orn^8^	Ile^10^	Average from Orn^8^/Ile^10^
**1a**	75	72	64	68
**1b**	17	36	0	18
**1c**	54	56	15	35.5
**1d**	50	50	49	49.5

## Supplementary Materials

The following are available online: peptide and aza(peptide) characterization data; LC-MS chromatograms; tabulated ^1^H NMR data for all peptide and aza(peptide) analogs.
