# Supplementary material for: Aza-Amino Acids Disrupt β-Sheet Secondary Structures
Source: Molecules. 2019 May 18;24(10):1919. doi: 10.3390/molecules24101919 (PMC6572070; doi:10.3390/molecules24101919)
Supplement: Supplementary file 1 [file molecules-24-01919-s001.pdf]

SUPPORTING INFORMATION

Aza-amino acids disrupt β-sheet secondary structures

Michael McMechen, Evan Willis, Preston Gourville, and Caroline Proulx*

Department of Chemistry, North Carolina State University, Raleigh, NC 27695-8204, USA.

e-mail: cproulx@ncsu.edu

I. Peptide and azapeptide characterization data

Table S1. Peptide and azapeptide characterization data.

peptide	crude purity[a]	purity[b]	yield[c]	m/z (cald) $[M+1]^+$	m/z (obsd) $[M+1]^+$	retention time[a] (min)
**1a** H-RYVEVpGOKILQ-NH_2	68	>99	8	1415.9	1415.2	8.05
**1b** H-RYazaVEVpGOKILQ-NH_2	36	99	8	1416.9	1416.1	8.26
**1c** H-RYvEVpGOKILQ-NH_2	74	>99	28	1415.9	1415.2	8.42
**1d** H-RYazaGEVpGOKILQ-NH_2	43	>99	8	1374.8	1374.1	7.93
**1e** c[RYVEVpGOKILQpG]	78	97	18	1552.9	1552.1	7.93
**1f** H-RYVEVPGOKILQ-NH_2	64	>99	13	1415.9	1415.2	7.96

[a]Unless otherwise noted, analytical LCMS analyses were performed on a 5 μM, 150 x 4.6 mm C18 Vydac column from Mac-Mod Analytical, Inc (cat# 218TP5415) with a flow rate of 0.5 mL/min using a 12 min 5-95% linear gradient of MeCN (0.1% TFA) in water (0.1% TFA). The purity at 214 nm wavelength is reported in all cases. [b]RP-HPLC purity at 214 nm after purification. [c]Yields after purification by RP-HPLC are based on resin loading.

II. LCMS Chromatograms and NMR spectra

H-Arg-Tyr-Val-Glu-Val-D-Pro-Gly-Orn-Lys-Ile-Leu-Gln-NH_2 (**1a**):

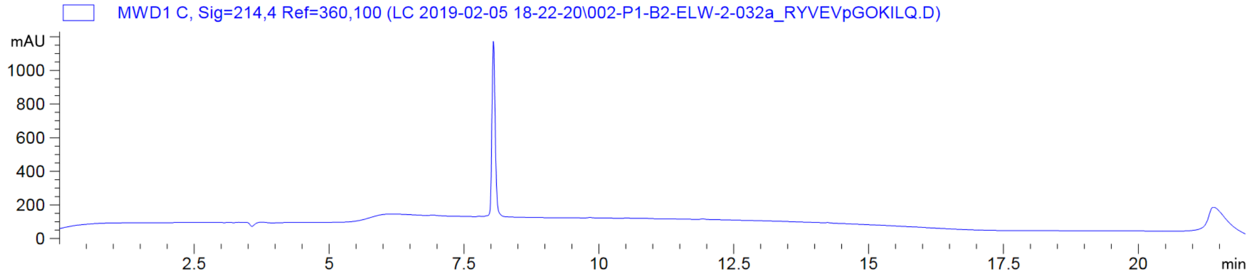

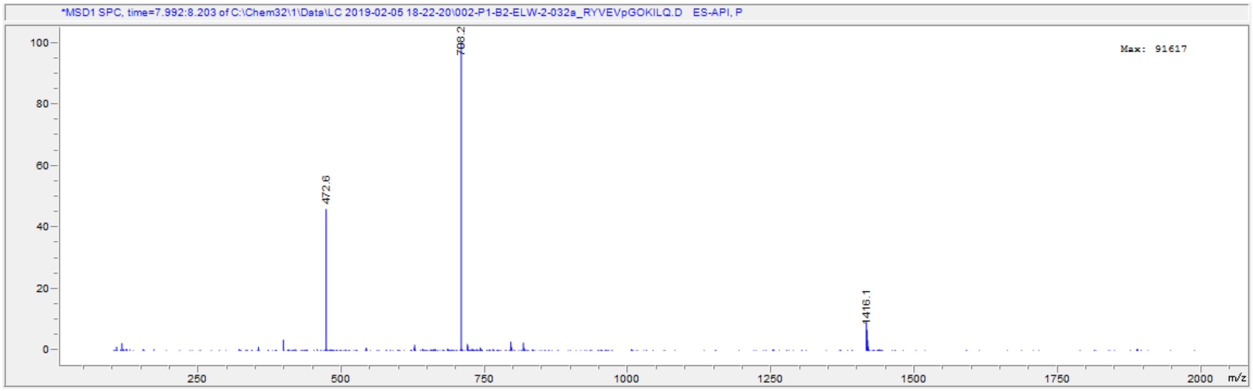

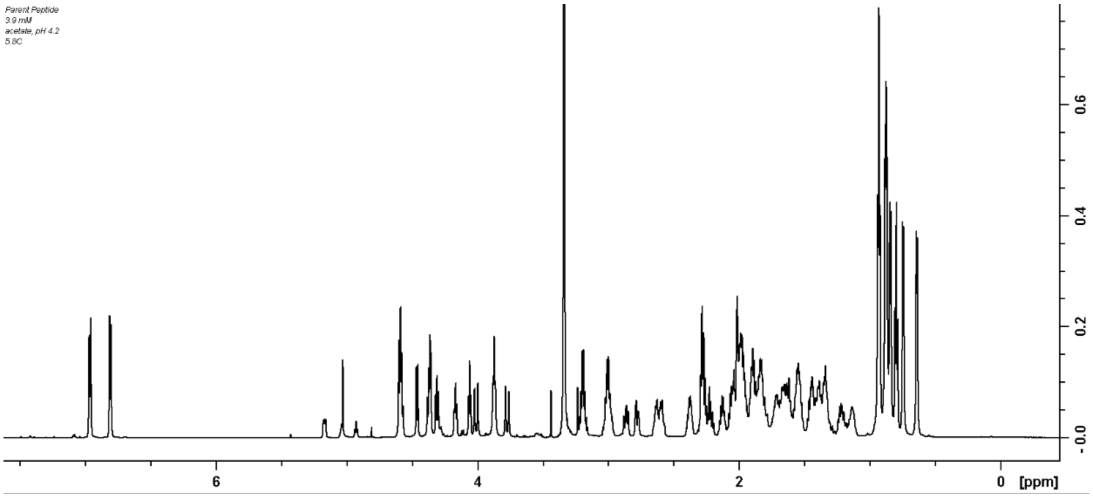

H-Arg-Tyr-azaVal-Glu-Val-Pro-Gly-Orn-Lys-Ile-Leu-Gln-NH_2 (**1b**):

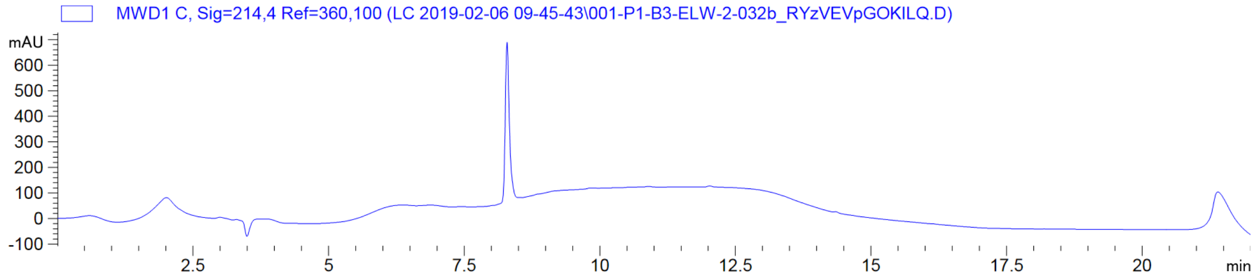

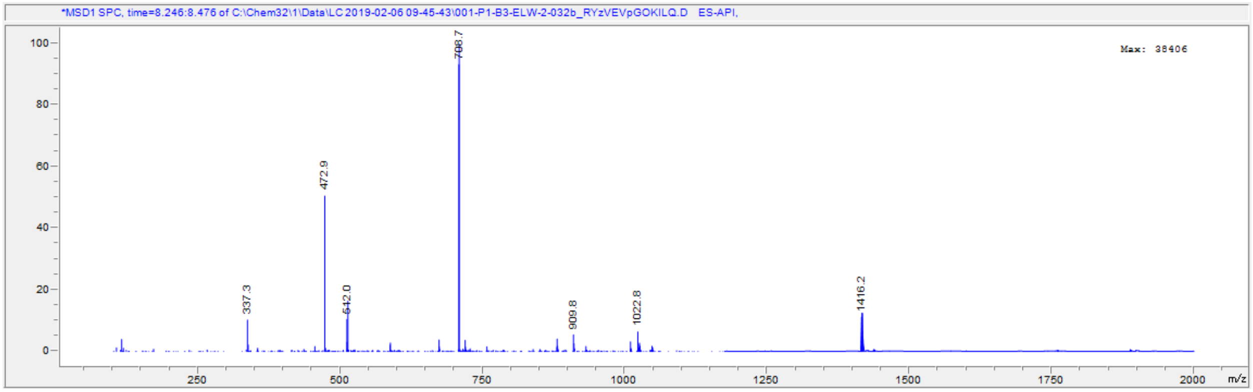

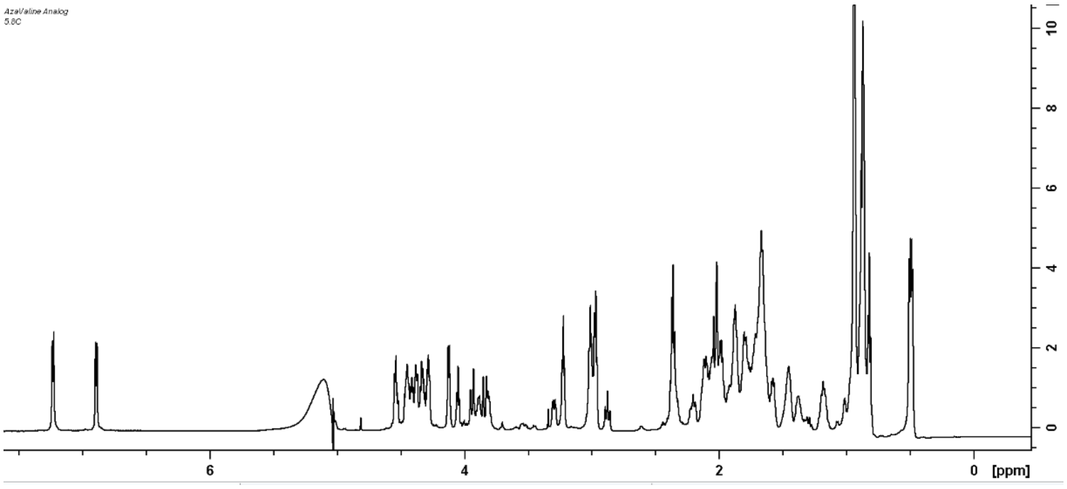

H-Arg-Tyr-D-Val-Glu-Val-Pro-Gly-Orn-Lys-Ile-Leu-Gln-NH_2 (**1c**):

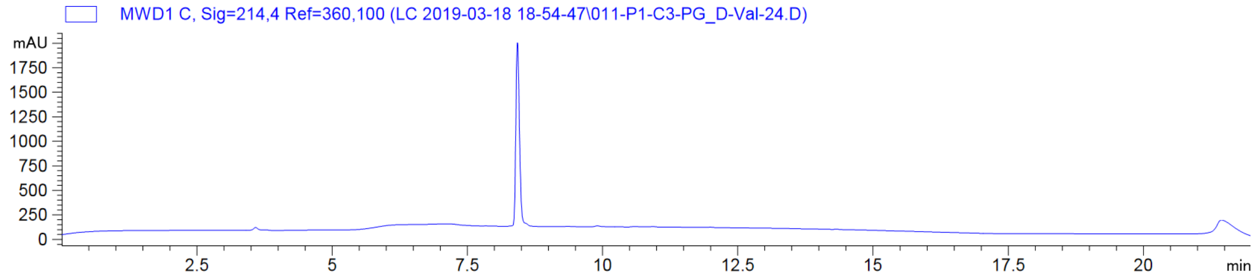

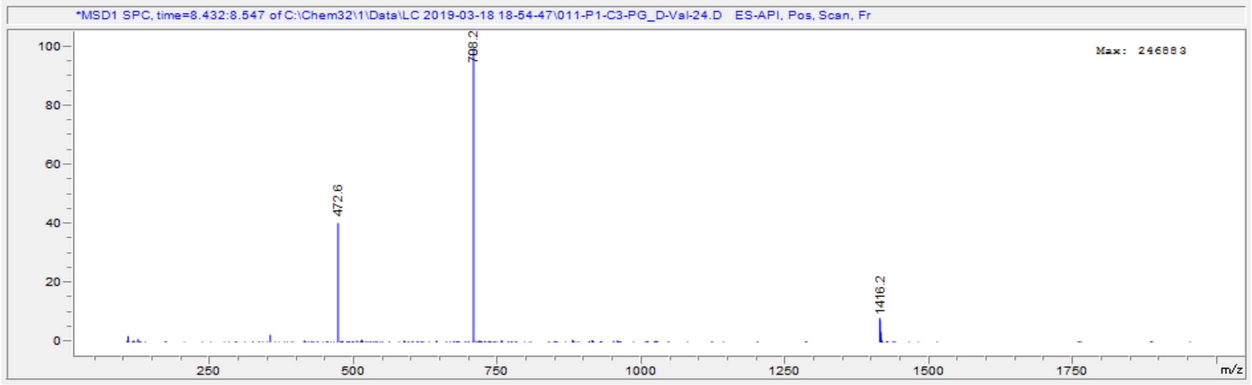

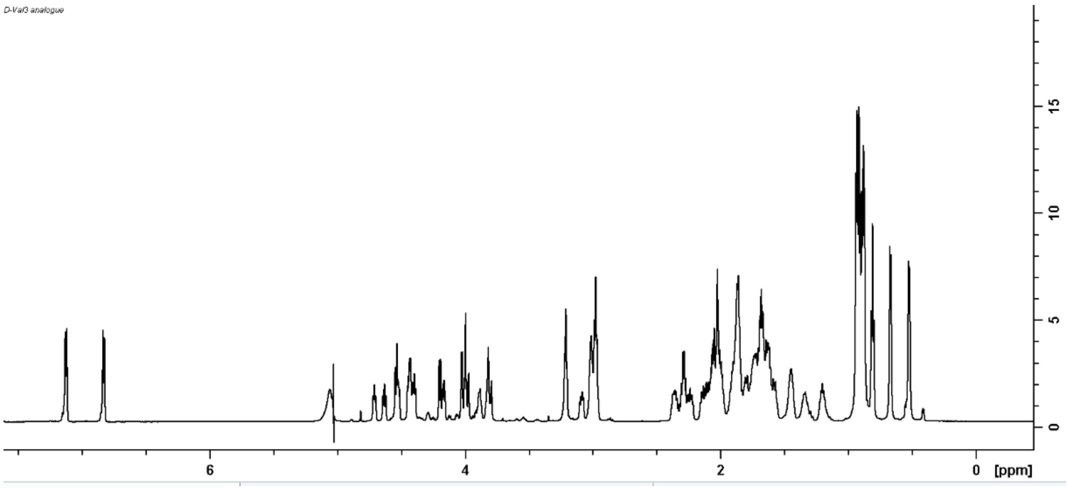

H-Arg-Tyr-azaGly-Glu-Val-Pro-Gly-Orn-Lys-Ile-Leu-Gln-NH_2 (**1d**):

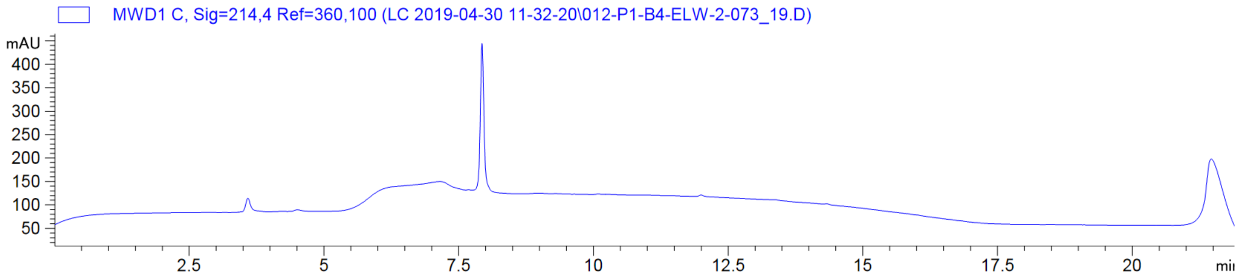

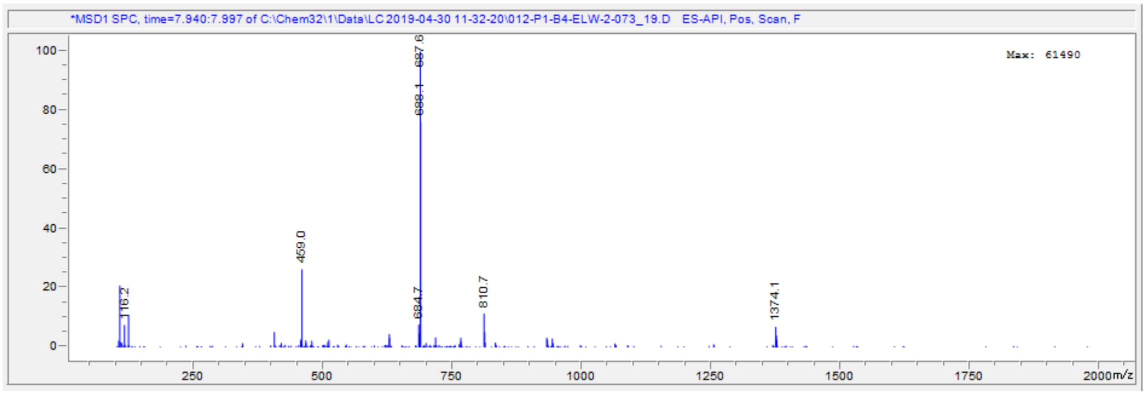

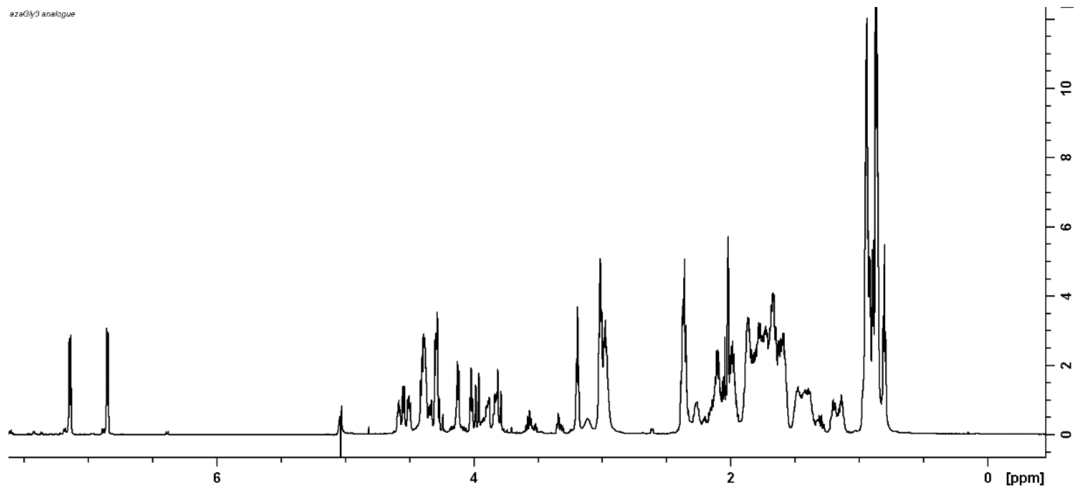

c[Arg-Tyr-Val-Glu-Val-D-Pro-Gly-Orn-Lys-Ile-Leu-Gln- D-Pro-Gly] (**1e**):

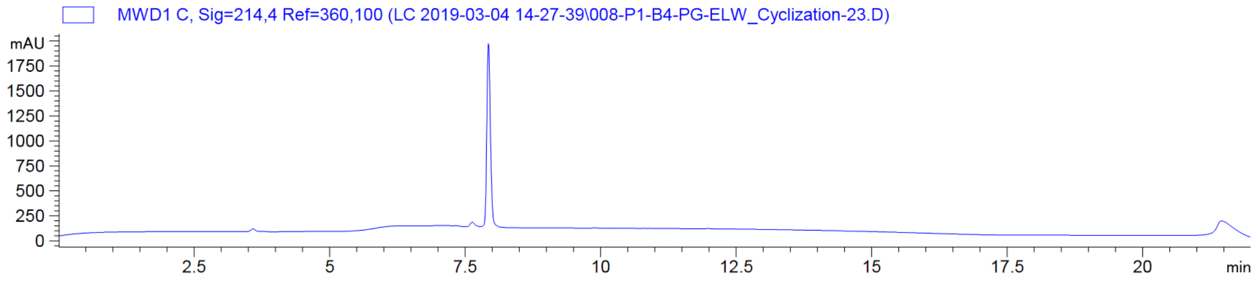

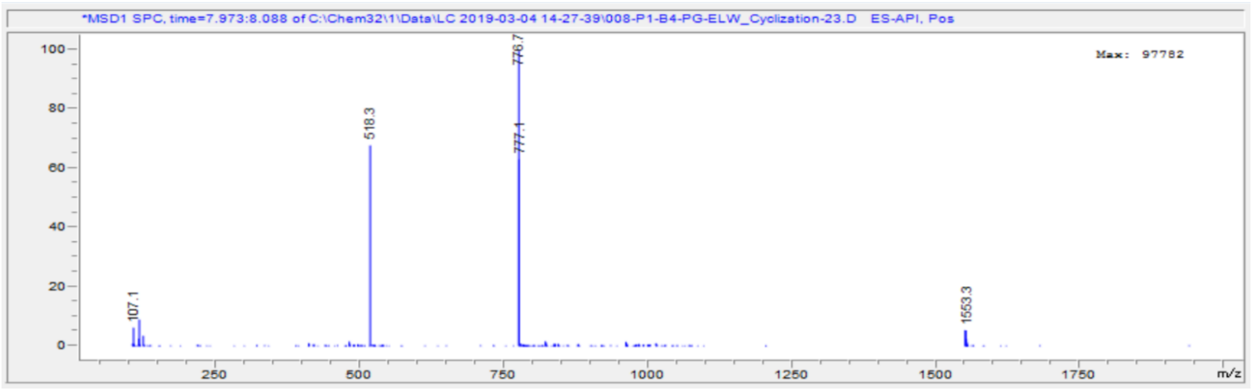

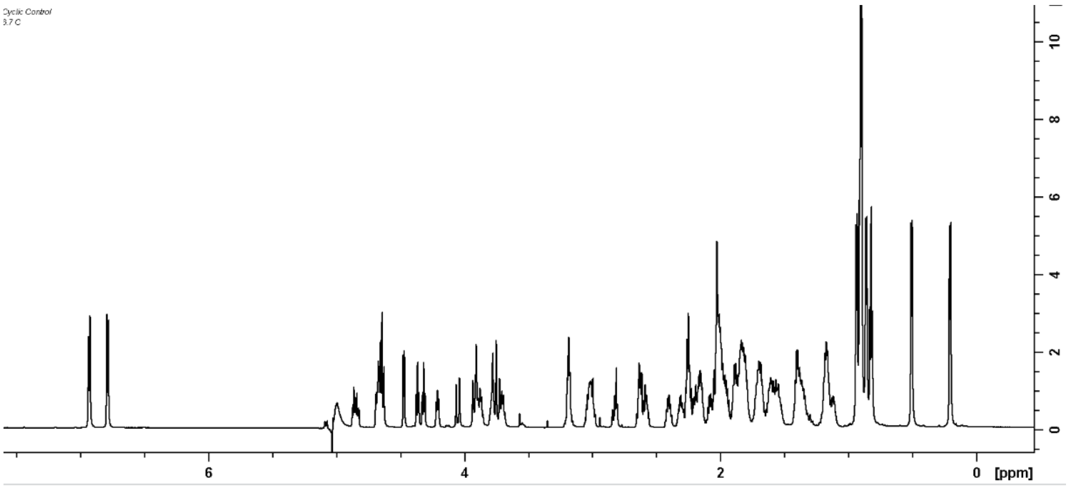

H-Arg-Tyr-Val-Glu-Val-Pro-Gly-Orn-Lys-Ile-Leu-Gln-NH_2 (**1f**):

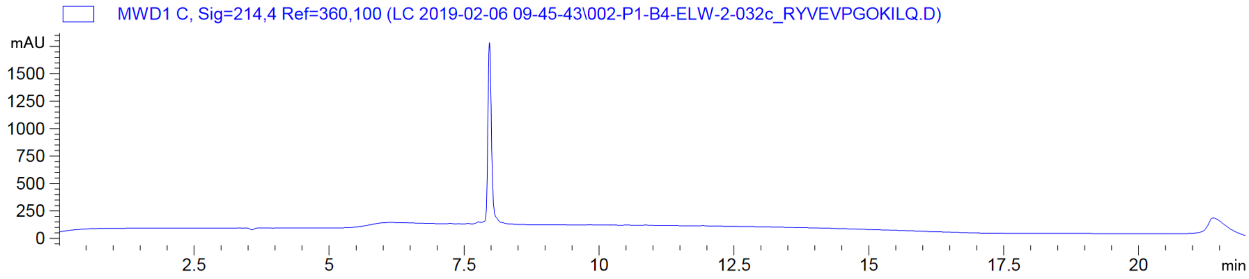

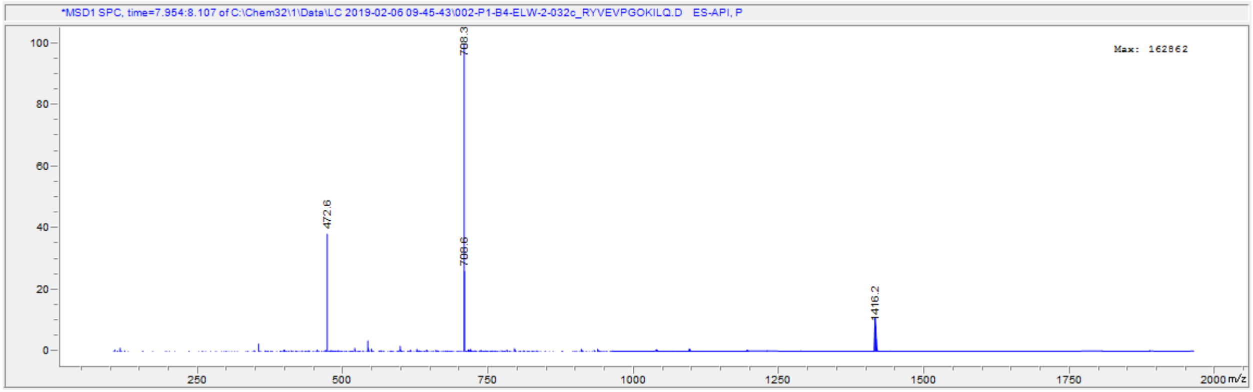

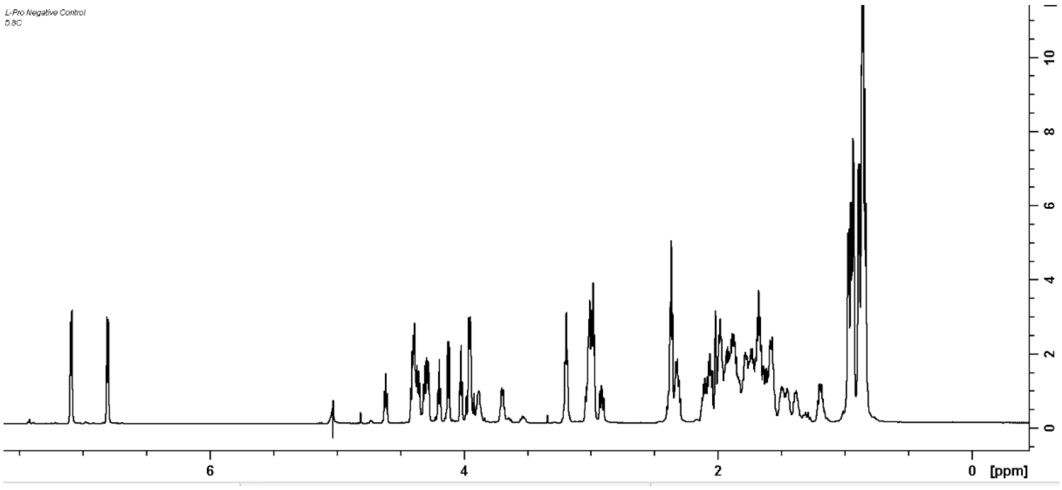

III. Tabulated ^{1}H NMR data

Chemical shifts (δ) are reported in ppm. All NMR data collected with the following conditions: 700 MHz, D_2O, pH = 4.2 (deuterated NaOAc buffer), 5.8°C. Proton assignments were made based on COSY, TOCSY, and ROESY spectra.

H-Arg-Tyr-Val-Glu-D-Pro-Gly-Orn-Lys-Ile-Leu-Gln-NH_2 (**1a**):

	α	β	Other:
Arg	4.06	1.92	γ CH_2: 1.55 δ CH_2: 3.19
Tyr	5.17	2.79, 2.87	2,6 H: 6.96 3,5 H: 6.81
Val^3	4.36	2.00	γ CH_3: 0.86
Glu	4.93	1.88, 1.97	γ CH_2: 2.24
Val^5	4.60	1.99	γ CH_3: 0.93
DPro	4.38	2.38	γ CH_2: 2.04, 2.12 δ CH_2: 3.87
Gly	3.78, 4.02	-	-
Orn	4.59	1.83	γ CH_2: 1.69, 2.12 δ CH_2: 3.00
Lys	4.58	1.54, 1.63	γ CH_2: 1.13 δ CH_2: 1.35 ε CH_2: 2.61
Ile	4.46	1.90	γ CH_2: 1.19, 1.48 γ CH_3: 0.88 δ CH_3: 0.80
Leu	4.17	1.62	γ CH_2: 1.45 δ CH_3: 0.64, 0.75
Gln	4.31	1.84, 2.04	γ CH_2: 2.29

H-Arg-Tyr-azaVal-Glu-Pro-Gly-Orn-Lys-Ile-Leu-Gln-NH_2 (**1b**):

	α	β	Other
Arg	4.05	1.88	γ CH_2: 1.65 δ CH_2: 3.22
Tyr	4.53	2.87, 3.29	2,6 H: 7.23 3,5 H: 6.89
azaVal^3	-	4.22	γ CH_3: 0.48
Glu	4.44	1.89	γ CH_2: 2.12, 2.19
Val^5	4.54	2.03	γ CH_3: 0.94
DPro	4.41	2.34	γ CH_2: 2.06, 2.33 δ CH_2: 3.80, 3.88
Gly	3.84, 3.94	-	-
Orn	4.46	1.78	γ CH_2: 1.71 δ CH_2: 3.00
Lys	4.34	1.72	γ CH_2: 1.37, 1.44 δ CH_2: 1.68, 1.75 ε CH_2: 2.97
Ile	4.12	1.80	γ CH_2: 1.46 γ CH_3: 1.18 δ CH_3: 0.87
Leu	4.38	1.67	γ CH_2: 1.56 δ CH_3: 0.86, 0.93
Gln	4.28	1.98	γ CH_2: 2.10, 2.36

H-Arg-Tyr- D-Val-Glu-Pro-Gly-Orn-Lys-Ile-Leu-Gln-NH_2 (**1c**):

	α	β	Other:
Arg	4.00	1.85	γ CH_2: 1.62 δ CH_2: 3.20
Tyr	4.64	2.96, 3.08	2,6 H: 7.13 3,5 H: 6.83
DVal^3	4.45	1.64	γ CH_3: 0.87, 0.93
Glu	4.71	1.89, 2.13	γ CH_2: 2.23
DVal^3	4.54	2.01	γ CH_3: 0.91
DPro	4.40	2.35	γ CH_2: 2.07 δ CH_2: 3.80, 3.88
Gly	3.80, 3.99	-	-

Orn	4.53	1.81	γ CH_2: 1.67 δ CH_2: 3.01
Lys	4.43	1.66	γ CH_2: 1.34, 1.44 δ CH_2: 1.73 ε CH_2: 2.96
Ile	4.20	1.87	γ CH_2: 1.20, 1.43 γ CH_3: 0.90 δ CH_3: 0.81
Leu	4.03	2.06	γ CH_2: 1.63 δ CH_3: 0.51, 0.65
Gln	4.17	1.84, 2.02	γ CH_2: 2.28

H-Arg-Tyr- azaGly-Glu-Pro-Gly-Orn-Lys-Ile-Leu-Gln-NH_2 (**1d**):

	α	β	Other
Arg	4.02	1.86	γ CH_2: 1.59 δ CH_2: 3.19
Tyr	4.55	2.64, 2.76	2,6 H: 7.14 3,5 H: 6.85
azaGly[3]	-	-	-
Glu	4.58	1.84	γ CH_2: 2.15, 2.26
Val[5]	4.54	2.01	γ CH_3: 0.93
DPro	4.40	2.35	γ CH_2: 1.98, 2.07 δ CH_2: 3.82, 3.89
Gly	3.80, 3.98	-	-
Orn	4.51	1.81	γ CH_2: 1.71 δ CH_2: 3.01
Lys	4.29	1.68	γ CH_2: 1.41 δ CH_2: 1.13 ε CH_2: 2.97
Ile	4.38	1.65, 1.73	γ CH_2: 1.38, 1.47 γ CH_3: 0.92 δ CH_3: 0.85
Leu	4.12	1.81	γ CH_2: 1.18 δ CH_3: 0.80, 0.87
Gln	4.29	1.97	γ CH_2: 2.10, 2.37

c[Arg-Tyr-Val-Glu-D-Pro-Gly-Orn-Lys-Ile-Leu-Gln- DPro-Gly] (**1e**):

	α	β	Other
Arg	4.65	1.82	γ CH_2: 1.53, 1.63 δ CH_2: 3.18
Tyr	5.08	2.82	2,6 H: 6.93 3,5 H: 6.79
Val[3]	4.48	2.03	γ CH_3: 0.87
Glu	5.00	1.88, 2.01	γ CH_2: 2.25
Val[5]	4.64	1.97	γ CH_3: 0.91
DPro[6]	4.37	2.00, 2.41	γ CH_2: 2.09, 2.16 δ CH_2: 3.90
Gly[7]	3.76, 4.06	-	-
Orn	4.69	1.82	γ CH_2: 1.70 δ CH_2: 3.01
Lys	4.84	1.59, 1.69	γ CH_2: 1.17 δ CH_2: 1.39 ε CH_2: 2.59
Ile	4.65	1.87	γ CH_2: 1.17, 1.36 γ CH_3: 0.91 δ CH_3: 0.82
Leu	4.21	1.56	γ CH_2: 1.14 δ CH_3: 0.50, 0.19
Gln	4.87	1.83, 2.02	γ CH_2: 2.21
DPro[13]	4.31	1.95, 2.32	γ CH_2: 1.98, 2.15 δ CH_2: 3.69, 3.77
Gly[14]	3.74, 3.92	-	-

H-Arg-Tyr-Val-Glu-Pro-Gly-Orn-Lys-Ile-Leu-Gln-NH_2 (**1f**):

	α	β	Other
Arg	4.02	1.88	γ CH_2: 1.57 δ CH_2: 3.19
Tyr	4.61	2.91, 3.02	2,6 H: 7.09 3,5 H: 6.81
Val[3]	3.96	1.87	γ CH_3: 0.84
Glu	4.20	1.94	γ CH_2: 2.33

Val[5]	4.40	2.06	γ CH_3: 0.95
LPro	4.41	2.30	γ CH_2: 1.93 δ CH_2: 3.68, 3.86
Gly	3.98, 3.92	-	-
Orn	4.33	1.87	γ CH_2: 1.67, 1.74 δ CH_2: 3.01
Lys	4.31	1.73	γ CH_2: 1.37, 1.44 δ CH_2: 1.67 ε CH_2: 2.97
Ile	4.12	1.83	γ CH_2: 1.18, 1.49 γ CH_3: 0.87 δ CH_3: 0.82
Leu	4.38	1.64	γ CH_2: 1.55 δ CH_3: 0.87, 0.92
Gln	4.29	2.10, 1.96	γ CH_2: 2.35

IV. Representative example ^{1}H NMR proton assignment of peptide 1b

Assignments were made beginning with the identification of α-β proton pairs using COSY within the distinctive α-proton region of the spectra. Each pairing was subsequently grouped with their respective TOCSY correlations. TOCSY correlations and sequential proton positions were verified using COSY. Avoidance of redundant amino acids within the peptide sequence allowed for the facile identification of side-chain patterns and assignment of respective α-proton shifts. ROESY spectra were implemented to distinguish Val[3] from Val[5] using through-space interactions of the methylene contained in D-Pro. Sample data for the assignment of the azaVal[3] analog can be seen below.

H-Arg-Tyr- azaVal-Glu-Pro-Gly-Orn-Lys-Ile-Leu-Gln-NH_2 (**1b**): COSY

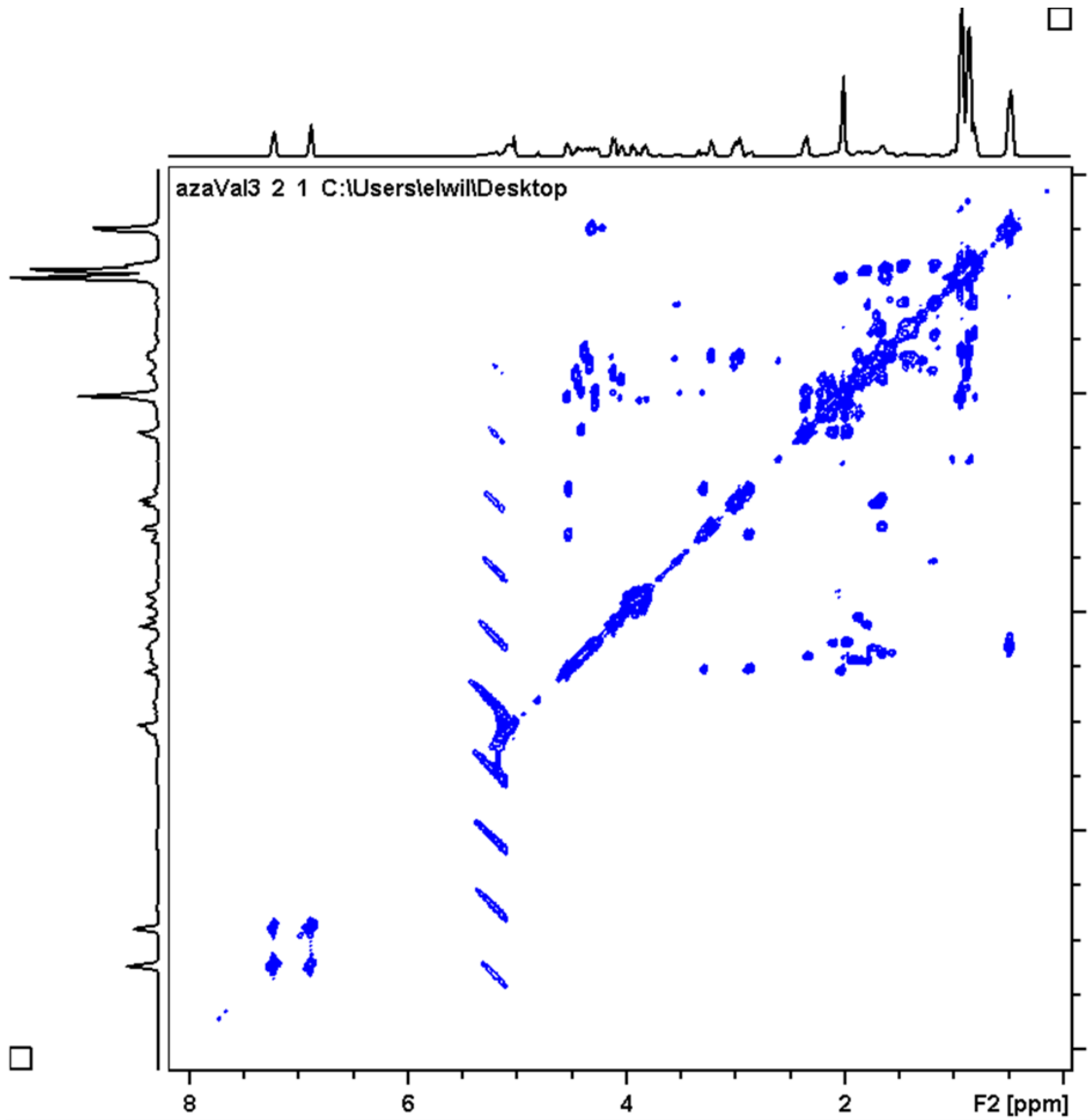

H-Arg-Tyr- azaVal-Glu-Pro-Gly-Orn-Lys-Ile-Leu-Gln-NH_2 (**1b**): COSY (zoom)

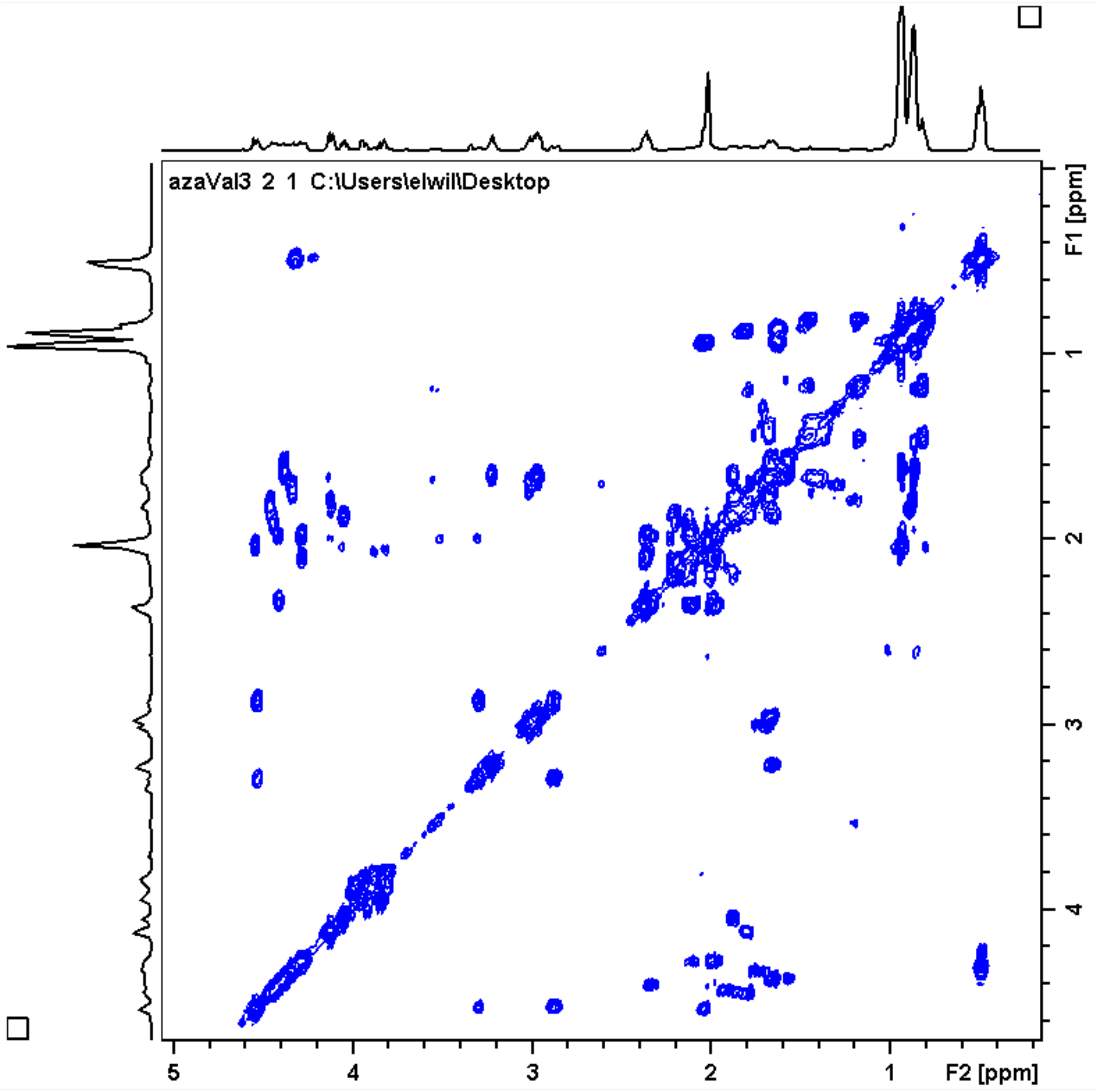

H-Arg-Tyr- azaVal-Glu-Pro-Gly-Orn-Lys-Ile-Leu-Gln-NH_2 (**1b**): TOCSY

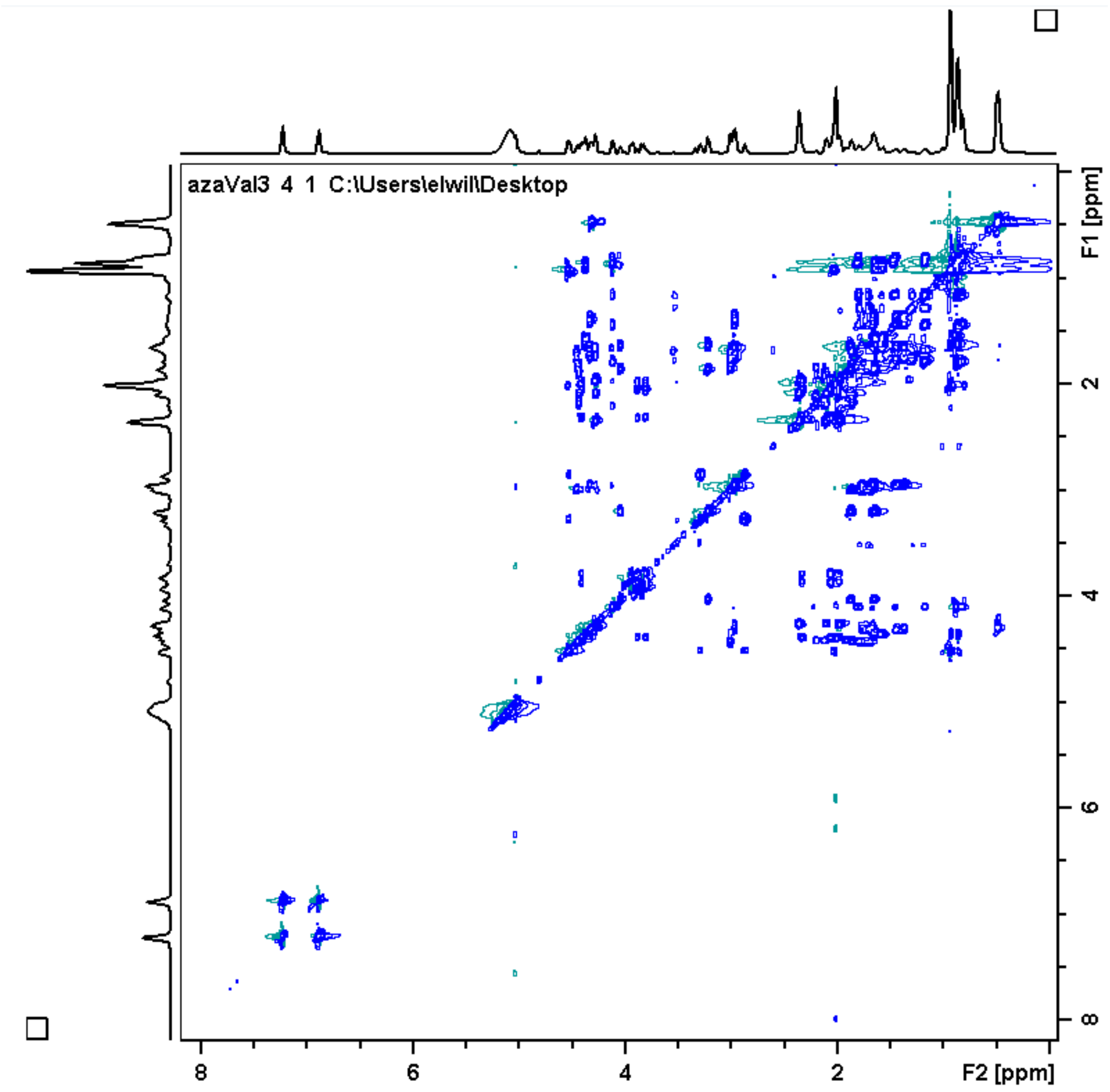

H-Arg-Tyr- azaVal-Glu-Pro-Gly-Orn-Lys-Ile-Leu-Gln-NH_2 (**1b**): TOCSY (zoom)

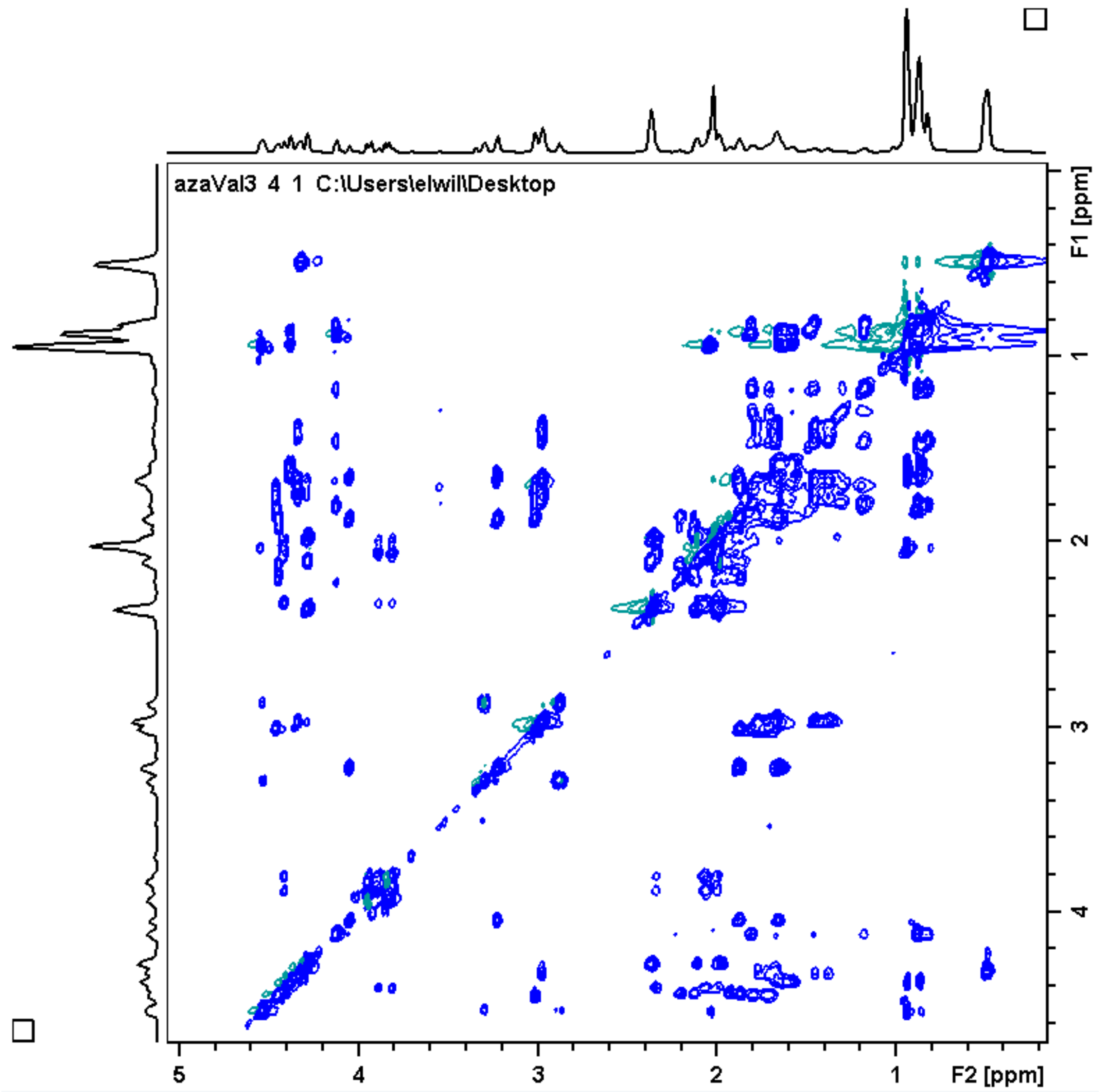

H-Arg-Tyr- azaVal-Glu-Pro-Gly-Orn-Lys-Ile-Leu-Gln-NH_2 (**1b**): ROESY

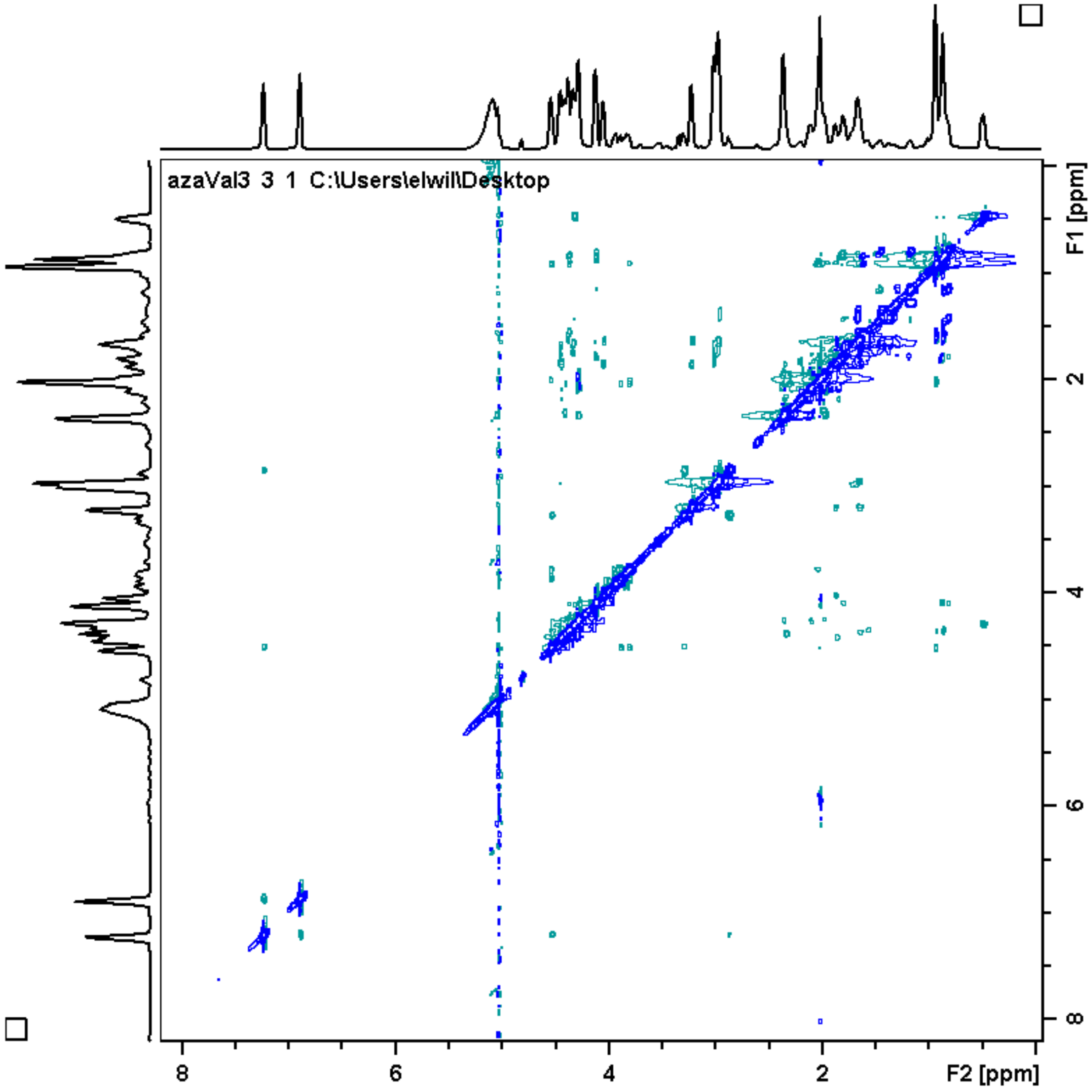

H-Arg-Tyr- azaVal-Glu-Pro-Gly-Orn-Lys-Ile-Leu-Gln-NH_2 (**1b**): ROESY (zoom)

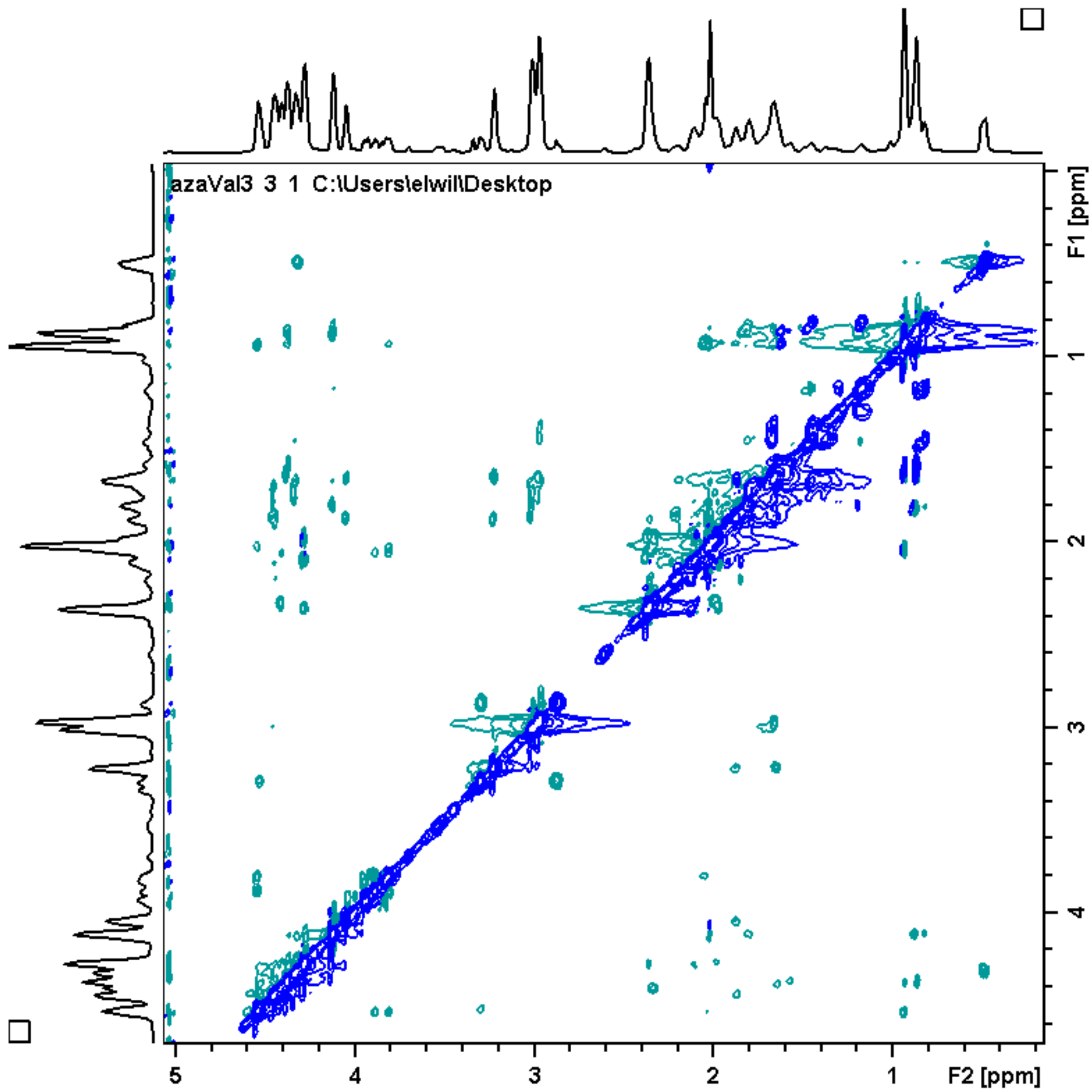
azaVal3 3 1 C:\Users\elwil\Desktop
F1 [ppm]
F2 [ppm]
1
2
3
4
5
